# Children’s self-reported exposure to sugary beverage advertisements and association with intake across six countries before and during the COVID-19 pandemic: a repeat cross-sectional study

**DOI:** 10.1186/s12889-024-20210-8

**Published:** 2024-10-11

**Authors:** Élisabeth Demers-Potvin, Simone Lemieux, Rachel B. Acton, Tarra L. Penney, Gary Sacks, Christine M. White, Martin White, David Hammond, Lana Vanderlee

**Affiliations:** 1 Centre de recherche Nutrition, santé et société (NUTRISS), Institut sur la nutrition et les aliments fonctionnels (INAF), Université Laval, 2440, Boulevard Hochelaga, Québec, G1V 0A6 Canada; 2 École de nutrition, Faculté des sciences de l’agriculture et de l’alimentation, Université Laval, Québec, Québec G1V 0A6 Canada; 3 School of Public Health Sciences, University of Waterloo, Waterloo, ON N2L 3G5 Canada; 4 School of Global Health, Faculty of Health, York University, Toronto, ON M3J 1P3 Canada; 5 School of Health & Social Development, Deakin University, Melbourne, VIC 3125 Australia; 6 MRC Epidemiology Unit, School of Clinical Medicine, Institute of Metabolic Science, University of Cambridge, Cambridge, CB2 0QQ UK

**Keywords:** Sugary drinks, Advertising, Marketing, Adolescents, Screen time, Online school classes

## Abstract

**Background:**

Children’s exposure to sugary beverage advertising may have changed during the COVID-19 pandemic due to shifts in media habits, which could subsequently have influenced intake. This study aimed to examine: 1) children’s frequency and setting of exposure to advertisements of sugary beverages in six countries before and during the COVID-19 pandemic; 2) the association between exposure to sugary beverage advertisements and intake.

**Methods:**

Children aged 10–17 years (*n* = 28,908) in Australia, Canada, Chile, Mexico, the United Kingdom (UK) and the United States (US) completed online surveys in 2019, 2020 and 2021 as part of the International Food Policy Study using a repeat cross-sectional study design. Respondents self-reported frequency and setting of exposure to sugary beverage advertisements, location of school classes (in-person/online, 2020–2021 only), screen time, and sugary beverage intake. Adjusted weighted logistic and negative binomial regression models stratified by country examined associations between year and reported sugary beverage advertising exposure, and associations between sugary beverage advertising exposure and intake. Differences in reported advertising exposure between students taking online or in-person school classes were explored.

**Results:**

Self-reported exposure to advertisements for sugary beverages *at least weekly* was relatively stable across years within countries, with differences in settings of exposure. Exposure to sugary beverage advertisements increased on digital media independently of screen time from 2019 to 2021 in Australia, Canada, the UK and US, with a concomitant decrease in exposure in retail settings in all countries except the UK. In Australia and the UK, children attending all classes online were more likely to report *at least weekly* (vs *less than once a week*) exposure to sugary beverage advertisements, and children attending all classes online were more likely to report exposure to advertisements on digital media and in other settings (*e.g.,* billboard, magazines) compared to children attending in-person classes in Australia, Canada and the UK. Exposure to sugary beverage advertisements *at least weekly* (IRR = 1.12,99%CI:1.09–1.15) and in each of the settings was associated with sugary beverage intake.

**Conclusions:**

Exposure to digital advertisements for sugary beverages increased from 2019 to 2021 in most countries, and exposure was associated with sugary beverage intake. Reducing children’s exposure to advertising of less healthy foods, including on digital media, may reduce sugary beverage intake.

**Supplementary Information:**

The online version contains supplementary material available at 10.1186/s12889-024-20210-8.

## Background

Children are exposed to high levels of food and beverage (hereafter, food) marketing [1–3]. Marketing encompasses a range of practices relating to price, product, place and promotion (e.g. advertising) [4], and is known to influence children’s preferences for products, consumption patterns, purchase requests to parents, and brand relationships that can extend into adulthood [5–9]. Children’s exposure to food marketing extends across a variety of media and settings [10], each employing diverse persuasive techniques that increase marketing effectiveness [11]. Digital marketing in particular has been shown to use persuasive techniques that are more effective among children [12, 13], which is of importance given children’s increasing use of digital platforms [14]. Children are often unaware of the persuasive intent of marketing, making them particularly vulnerable to its influence [15].

The majority of food marketing to which children are exposed promotes energy-dense and nutrient-poor (hereafter, less healthy) foods, including sugary beverages (*i.e*., beverages containing free sugars) [3, 16–18]. Recent studies identified sugary beverages among the foods with the highest advertising expenditures [19, 20]. Most sugary beverages contribute minimally to satiety and have a high content of free sugars, and thus may contribute to excess energy intake and weight gain, among other health conditions [21–26]. While evidence indicates reductions in children’s sugary beverage intake over the past decades [27–29], sugary beverages remain the main source of free sugars in children’s diets in many countries [30–32].

Comprehensive marketing restrictions to protect children from the harmful influence of less healthy food marketing are recommended by the World Health Organization (WHO) [11]. Jurisdictions worldwide, such as Chile, Mexico, the United Kingdom (UK) and the province of Québec (Canada), have implemented mandatory restrictions which apply to different media and settings [33]. Despite this, children continue to be highly exposed to less healthy food marketing as existing restrictions pertain mostly to advertising on television, and often apply in a limited way, for example where it is deemed that the target audience is children 12 years and younger [33].

The COVID-19 pandemic may have influenced children’s exposure to sugary beverage advertising. Containment and closure measures implemented by governments worldwide to decrease the transmission of the virus (mostly in 2020–2021) led to a shift to remote learning online and on television [34, 35], increasing children’s screen time [34, 36]. As an increase in reported screen time has been associated with more frequent reported exposure to sugary beverage advertising among children [37], this may have increased exposure to advertising of sugary beverages, and subsequently, their intake [5].

We therefore designed this study to examine children’s self-reported frequency and setting of exposure to advertisements of sugary beverages across six high and upper-middle income countries (Australia, Canada, Chile, Mexico, UK, United States [US]) before (2019) and during (2020–2021) the COVID-19 pandemic. We also aimed to examine the association between children’s self-reported exposure to sugary beverage advertisements and intake of sugary beverages in the same countries and years.

## Methods

### Study design, sampling and recruitment

Data were analyzed from the 2019, 2020, and 2021 International Food Policy Study (IFPS) Youth Surveys, annual repeat cross-sectional surveys conducted in Australia, Canada, Chile, Mexico, the UK and the US. These countries have different policies regarding restrictions on advertising of less healthy foods to children, with Chile and Mexico having more stringent policies on television and the UK restricting less healthy food advertising on television during children’s programs. In Australia, Canada and the US, industry-led self-regulatory approaches are used [33]. Children’s sugary beverage intake patterns in these countries are also known to differ [27], and these countries differed in the timing of school closures due to COVID-19 [34, 35].

Data were collected via self-completed web-based surveys conducted in November–December 2019 (before COVID-19), 2020 and 2021 (during COVID-19) with children aged 10 to 17 years. In its latest guideline on less healthy food marketing restrictions, the WHO defines children as any person under the age of 18 years, in line with the Convention on the Rights of the Child definition [11]; the term “children” in this paper is applied accordingly. Respondents were recruited through parents/guardians enrolled in the Nielsen Consumer Insights Global Panel and their partners’ panels. Email invitations with unique survey links were sent to adult panelists within each country. Those who confirmed they had a child aged 10 to 17 living in their household were asked for permission for their child to complete the survey (only one child per household was invited). Children aged 10 to 17 years were eligible to participate, with quotas for age and sex groups in the UK and US (quotas were not applied in other countries due to smaller panel sizes). After eligibility screening, all potential child respondents were provided with information about the study and asked to provide assent. Surveys were conducted in English in Australia and the UK; Spanish in Chile and Mexico; English or French in Canada; and English or Spanish in the US. Members of the research team who were native in each language reviewed the French and Spanish translations independently. The child’s parent/guardian received remuneration in accordance with their panel’s usual incentive structure. The median survey time was 24 min (2019), 25 min (2020) and 22 min (2021). The participation rate (*i.e.,* the number of respondents who completed the survey among the total number of invites) was 1.5% in 2019, 3.5% in 2020, and 4.7% in 2021. The cooperation rate (*i.e.,* the percentage of respondents who completed the survey among those eligible who accessed the survey link) was 76.8% (2019), 79.6% (2020) and 81.9% (2021). A full description of the study methods can be found in the IFPS Youth Survey Technical Report [38], and each wave of the IFPS survey is available online [39].

### Variables used in the analysis

#### Frequency of exposure to sugary beverage advertisements

Self-reported frequency of exposure to sugary beverage advertisements was assessed using the question: “*In the last 30 days, how often did you see or hear advertisements for these kinds of food or drinks?”* Participants responded for six food categories; the current analysis examined only the measure for sugary beverages. (Response options: ‘never’, ‘less than once a week’, ‘once a week’, ‘a few times a week’, ‘everyday’, ‘more than once a day’, ‘don’t know’ and ‘refuse to answer’). A binary variable was coded to prioritize reporting of overall trends and minimize a focus on precise reporting, and to facilitate interpretation of results. Responses of ‘once a week’, ‘a few times a week’, ‘everyday’ and ‘more than once a day’ were combined as *at least weekly* exposure. ‘Never’ and ‘less than once a week’ were combined as *less than once a week* exposure. Responses of ‘don’t know’ and ‘refuse to answer’ were excluded from analyses (*n* = 2592).

#### Setting of exposure to sugary beverage brand advertisements

To assess self-reported setting of exposure to sugary beverage brand advertisements, respondents were shown three logos of sugary beverage brands popular in all IFPS countries, as per market share for off-trade sales volumes [40] of regular soda (Coca-Cola), energy drink (Red Bull) and juice (country-specific brands) and were asked “*Have you seen any advertisements for this drink brand in the last 30 days?*” (Response options: ‘yes’, ‘no’, ‘don’t know’, and ‘refuse to answer’). Responses of ‘don’t know’ were recoded as ‘no’. ‘Refuse to answer’ responses were excluded from analyses (*n* = 32). A technical glitch occurred for *n* = 36 respondents; they were thus removed from analyses. If a respondent had an affirmative response to at least one brand, they were randomized to one of the brands for which they had seen an advertisement (or shown the only brand they reported to have seen) and were asked: “*Where did you see advertisements for this brand?*” Respondents were asked to select all the settings of exposure to brand advertisements from a list of 14 settings which were collapsed into four categories: 1) Television (‘Television shows, series or movies’); 2) Digital media (‘Website or social media’, ‘Video or computer games’); 3) Retail settings and promotions (‘Stores’, ‘Contests, free samples or coupons’, ‘Movie theatres’); 4) Other settings (‘Radio’, ‘Magazine or newspaper’, ‘Billboard’, ‘Buses, bus stops and other public transit’, ‘School’, ‘Recreation or community centre’, ‘Sports event, concert or community event’, ‘Other [Open text]’). Responses for all brands were merged to derive the setting of exposure to a sugary beverage advertisement. For example, exposure to Coca-Cola, Red Bull and juice brand advertisements on television were merged as *exposure to advertisements of sugary beverages on television*. Responses of ‘don’t know’, ‘refuse to answer’ and discordant answers were removed from the sample (*n* = 660). Open text responses were reviewed and recoded to be included in one of the setting categories above (when applicable; *n* = 529).

#### Sugary beverage intake

Sugary beverage intake was assessed using the online Beverage Frequency Questionnaire (BFQ) adapted from the adult version following cognitive testing among children aged 10–13 years (unpublished data). A list of 14 different beverage categories was shown with examples of beverages for each category (Table 1), and respondents were asked “*Did you drink any of these in the last 7 days*?” (Response options: ‘yes’, ‘no’, ‘don’t know’ or ‘refuse to answer’). For each affirmative response, respondents were asked “*How many of these drinks did you have in the last 7 days*?” (Response options: A drop-down list of 0 to 20 drinks, ‘more than 20’, ‘don’t know’, ‘refuse to answer’). ‘More than 20’ was recoded as ‘21’. Participants who responded ‘yes’ to having consumed a category of beverage, but ‘0’ for the number of times they consumed it were recoded as not having consumed any, and responses of ‘no’ were recoded as ‘0’ for the number of times the beverage category was consumed.

**Table 1:** Beverage categories and examples of beverages in the adapted Beverage Frequency Questionnaire

Beverage category	Examples of beverages
Regular soda/pop/soft/fizzy drinks	Coke, Pepsi, 7-up, Sprite
Diet soda/pop/soft/fizzy drinks	Diet Pepsi, Coke Zero
Sports drinks	Gatorade, Powerade
Energy drinks	Red Bull, Rockstar, Monster
Frozen drinks	Slurpees, slushies, Slush Puppies
Coffee or tea with sugar	Lattes, mochas, frappuccinos, iced cappuccinos with sugar
Coffee or tea with no sugar	Lattes, mochas, frappuccinos, iced cappuccinos with no sugar
100% fruit or vegetable juice	Orange juice, apple juice
Fruit drinks	Lemonade, iced tea, SunnyD, fruit punch/cocktail, coconut water
Flavoured waters or vitamin waters	Crystal Light, Vitaminwater, Nestle Splash, squash
Water	Tap, bottled or sparkling water
Smoothies, protein shakes or drinkable yogurt	
White/dairy milk or alternatives like unsweetened soy or almond milk	
Chocolate or flavoured milk, hot chocolate or alternatives like sweetened soy or almond milk	

A sugary beverage intake variable was created by summing the number of beverages that contained free sugars: regular soda/pop, sports drinks, energy drinks, frozen drinks, coffee/tea with sugar, 100% fruit/vegetable juice, fruit drinks, flavoured waters/vitamin waters, smoothies, protein shakes/drinkable yogurt, and chocolate/flavoured milk. As some of these beverages may have been sweetened with non-sugar sweeteners, older children aged 14–17 years were asked to report the number of sports drinks, energy drinks, fruit drinks and flavoured waters that were ‘diet, low-calorie or no-calorie’. Sensitivity analyses were performed by subtracting the number of ‘diet, low-calorie or no-calorie’ beverages from the reported number of sugary beverages consumed among older children. Winsorization was performed to limit the impact of extreme values on the total number of sugary beverages consumed, with 70 sugary beverages per week (*i.e.,* 10 sugary beverages per day, all sugary beverages combined) determined as the maximum value. Respondents’ total sugary beverage intake that exceeded this value was recoded to 70 (*n* = 87). Participants who selected ‘don’t know’ and/or ‘refuse to answer’ for the questions above for any category and those who selected all categories, or no category (including water) were excluded from analyses that included measures of sugary beverage intake (*n* = 2904).

#### Location of school classes during COVID-19

Location of school classes at the time of the survey was assessed in the 2020 and 2021 surveys using the question: “*In some places, school has changed because of COVID-19. Are you taking your classes at school or online/from home?”* (Response options: ‘all classes at school’, ‘all classes online/from home’, ‘some classes at school, some classes online/from home’, ‘don’t know’, and ‘refuse to answer’). Responses of ‘don’t know’ and ‘refuse to answer’ were excluded from analyses (*n* = 669).

#### Total screen time

Total screen time was derived using the question: “On a normal weekday, how much time do you spend…” which listed five different types of media: ‘Watching YouTube’, ‘On social media’, ‘Watching TV shows, series or movies’, ‘Playing games on smartphones, computers, or game consoles’, ‘Browsing, reading websites, Googling, etc.’ Respondents selected time spent on each media using a scale from ‘0 h’ to ‘More than 4 h’, with intermediate options. ‘Don’t know’ and ‘refuse to answer’ responses for any media were excluded from analyses (*n* = 674). Responses for each media were recoded in hours and summed to estimate total screen time, which did not account for viewing multiple screens simultaneously. Extreme values of total screen time were winsorized to the mean + 2SD for each survey year (2019: 1192 min; 2020: 1228 min; 2021: 1173 min). A full description of the recoding of this variable is available elsewhere [37].

#### Sociodemographic measures

Sociodemographic characteristics included age category (10–12 years, 13–17 years), sex at birth (male, female), ethnicity category (assessed using census questions adapted from each country and recoded to ‘majority’ or ‘minority’ to allow comparisons across countries [description of recoding in Table 2]) and perceived income adequacy (not enough money, barely enough money, enough money, more than enough money). Full sociodemographic survey measures are available elsewhere [39].

#### Data analysis

A total of 34,874 children completed the surveys across the six countries in 2019, 2020 and 2021. Respondents were excluded for the following reasons: ineligible region (*i.e.,* failed to state their region or report a region in another country); invalid response to a data quality question; below minimum survey completion time based on median survey time; and/or multiple invalid responses to open-ended measures (*n* = 1276; 3.7%). Among included respondents, those with missing data (‘don’t know’, ‘refuse to answer’, technical glitch in the survey, discordant or implausible responses) for outcome variables and covariates were excluded from analyses. Two different analytic samples were created for the two main objectives of this paper. For analyses with frequency of exposure to sugary beverage advertisements as the outcome, the sample size was *n* = 28,908. For analyses examining sugary beverage intake, the sample size was *n* = 26,465 (removing those with missing data for sugary beverage intake measures).

Data were weighted with post-stratification sample weights constructed using a raking algorithm with population estimates from the census in each country based on age group, sex, region, and ethnicity (except in Canada). Estimates reported are weighted and 99% confidence intervals (CI) are reported for adjusted odds ratios (AOR) and incidence rate ratios (IRR). Analyses were conducted using SAS OnDemand for Academics (SAS Institute Inc., North Carolina).

Unadjusted descriptive statistics (frequencies) stratified by country examined frequency of exposure to sugary beverage advertisements and settings of exposure to sugary beverage brand advertisements.

An overall model was used to test whether changes over time in reported frequency of exposure to sugary beverage advertisements differed by country. As the interaction between country and year was significant (*p*-value < 0.01), subsequent models were stratified by country. Separate binary logistic regression models examined differences in the odds of reporting *at least weekly* exposure to sugary beverage advertisements across years, within countries. Models were adjusted for sex, age, ethnicity, perceived income adequacy and total screen time, as this variable has been identified in the literature as being associated with self-reported exposure to advertisements of sugary beverages in children [37]. An additional binary logistic regression model in a sub-sample (2020 and 2021 only) examined the association between location of school classes during the COVID-19 pandemic and frequency of exposure to sugary beverage advertisements, adjusting for year and the same set of variables. The model was stratified by country as the interaction between country and location of school classes was significant (*p* < 0.01). Sensitivity analyses tested a series of univariate regression models to explore if total screen time mediated the association [41].

Overall models examined whether variations in the reported setting of exposure to sugary beverage brand advertisements differed across countries over years. The interaction between country and year was significant (*p*-value < 0.01), thus models were stratified by country. Separate binary logistic regression models stratified by country were computed to assess the likelihood of self-reporting exposure to sugary beverage brand advertisements in four different settings across years. Models were adjusted for the same variables as above (adjusted for total screen time only in models with television and digital media as the outcome), as well as the beverage brand to which the respondent was randomized. Additional models in a sub-sample (2020 and 2021 only) examined the association between location of school classes during the COVID-19 pandemic and setting of exposure to sugary beverage brand advertisements (stratified by country), adjusting for year and the same set of variables described above. Sensitivity analyses tested a series of univariate regression models to explore if total screen time mediated the association between location of school classes and setting of advertising exposure (only for television and digital media) [41].

A negative binomial regression examined the association between sugary beverage advertisement exposure variables (frequency and setting) and intake of sugary beverages using a count variable of sugary beverages consumed in the past seven days, adjusting for country, year and the same set of variables. To identify if location of school classes during the COVID-19 pandemic was a moderator of the association between exposure to sugary beverage advertisements and sugary beverage intake, interactions between advertisement exposure variables and location of school classes were tested, with interactions considered significant at *p*-values < 0.01.

## Results

### Sample characteristics

Weighted characteristics of the overall sample and by country are presented in Table 2. The sample size in Canada was larger than in Australia, Chile, Mexico, the UK and the US. The majority of the sample in each country was 13–17 years old and reported higher perceived income adequacy (‘Enough money’ or ‘More than enough money’). Distribution of ethnicity categories varied across countries, with a relatively smaller proportion of US respondents in the ‘majority’ category.

**Table 2:** Weighted characteristics among the overall sample and across six countries in 2019, 2020, 2021

Characteristic	Overall (*N*=28 908)	Australia (*n*=3321)	Canada (*n*=9009)	Chile (*n*=3618)	Mexico (*n*=4661)	UK (*n*=3864)	US (*n*=4435)
	% (n)						
**Year**							
2019	34 (9753)	37 (1243)	34 (3085)	32 (1153)	33 (1534)	33 (1288)	33 (1450)
2020	36 (10282)	40 (1330)	34 (3081)	41 (1472)	36 (1684)	33 (1277)	32 (1438)
2021	31 (8873)	23 (748)	32 (2843)	27 (993)	31 (1443)	34 (1299)	35 (1547)
**Age (years)**							
10-12	37 (10630)	38 (1253)	38 (3432)	35 (1282)	36 (1689)	37 (1413)	35 (1560)
13-17	63 (18278)	62 (2068)	62 (5577)	65 (2336)	64 (2972)	63 (2451)	65 (2875)
**Sex**							
Male	51 (14803)	51 (1709)	51 (4623)	51 (1847)	51 (2368)	51 (1990)	51 (2266)
Female	49 (14105)	49 (1612)	49 (4386)	49 (1771)	49 (2293)	49 (1874)	49 (2169)
**Ethnicity**							
Majority	73 (21175)	75 (2476)	71 (6402)	86 (3099)	80 (3706)	83 (3195)	52 (2297)
Minority	27 (7733)	25 (845)	29 (2607)	14 (519)	20 (955)	17 (669)	48 (2138)
**Perceived Income Adequacy**							
Not enough money	4 (1119)	4 (146)	2 (225)	5 (186)	4 (195)	4 (155)	5 (196)
Barely enough money	20 (5890)	17 (571)	15 (1367)	24 (876)	28 (1313)	19 (746)	23 (1017)
Enough money	62 (17988)	63 (2094)	63 (5661)	65 (2356)	62 (2911)	64 (2463)	56 (2503)
More than enough money	14 (3911)	15 (510)	19 (1756)	6 (200)	5 (242)	13 (500)	16 (703)

*Abbreviations:* *UK* United Kingdom, *US* United States.Ethnicity categories as per measures drawn from government-led national surveys in each country and recoded as follows: 1) Australia majority=only speaks English at home, minority=speaks a language other than English at home; 2) Canada majority=White, minority=other ethnicity; 3) Chile majority=Non-indigenous, minority=indigenous; 4) Mexico majority=Non-indigenous, minority=indigenous; 5) UK majority=White, minority=other ethnicity; 6) US majority=White, minority=other ethnicity

### Frequency of exposure to sugary beverage advertisements

**Fig. 1:**
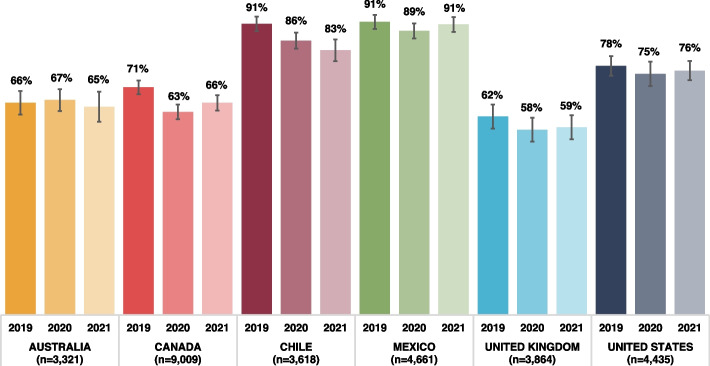
Percentage of respondents who reported *at least weekly* exposure to sugary beverage advertisements (*N* = 28 908)

Figure 1 presents the unadjusted weighted percentages of respondents who reported *at least weekly* exposure to sugary beverage advertisements by country in 2019, 2020 and 2021. Overall, 58% to 91% of respondents reported *at least weekly* exposure to sugary beverage advertisements, which varied by country and year, with the greatest percentages in Chile and Mexico. Respondents’ reported levels of frequency of exposure to sugary beverage advertisements are shown in Additional File 1. In general, exposure *a few times a week* was the most frequent response in all countries. A smaller proportion of respondents in Chile and Mexico reported *never* being exposed to sugary beverage advertisements and a greater proportion of respondents in Chile, Mexico and the US reported exposure *more than once a day*.

Table 3 presents estimates from separate country-specific binary logistic regression models examining differences in the odds of respondents reporting *at least weekly* exposure to sugary beverage advertisements between years. Children in Canada and Chile were less likely to report *at least weekly* exposure to sugary beverage advertisements in 2020 and 2021 compared to 2019, and children in Canada were more likely to report *at least weekly* exposure in 2021 compared to 2020. No differences were observed in *at least weekly* reported exposure to advertisements of sugary beverages between years in Australia, Mexico, the UK and the US.

**Table 3:** Estimates from logistic regression models examining children's exposure to sugary beverage advertisements *at least weekly*

Parameter	Australia (*n* = 3321)	Canada (*n* = 9009)	Chile (*n* = 3618)	Mexico (*n* = 4661)	United Kingdom (*n* = 3864)	United States (*n* = 4435)
	**AOR (99% CI)**	**AOR (99% CI)**	**AOR (99% CI)**	**AOR (99% CI)**	**AOR (99% CI)**	**AOR (99% CI)**
**Year**						
2019 vs 2020	1.05 (0.83, 1.32)	0.69 (0.59, 0.79)	0.57 (0.40, 0.80)	0.72 (0.50, 1.03)	0.83 (0.67, 1.04)	0.89 (0.68, 1.16)
2019 vs 2021	0.95 (0.73, 1.24)	0.81 (0.70, 0.94)	0.47 (0.32, 0.67)	0.91 (0.61, 1.34)	0.88 (0.70, 1.10)	0.96 (0.75, 1.22)
2020 vs 2021	0.91 (0.70, 1.18)	1.18 (1.02, 1.36)	0.82 (0.60, 1.13)	1.26 (0.88, 1.80)	1.06 (0.85, 1.32)	1.08 (0.83, 1.40)

Models were adjusted for: sex, age category, ethnicity, perceived income adequacy and total screen time

The year listed first is the reference variable. *N* = 28 908

*Abbreviations*: *AOR* Adjusted odds ratio, *CI* confidence interval

### Location of school classes during COVID-19 and frequency of exposure to sugary beverage advertisements

Separate binary logistic regression models examined the association between location of school classes during 2020–2021 and frequency of exposure to sugary beverage advertisements in each country (Additional file 2). Respondents in Australia and the UK who were taking all classes online/from home at the time of the surveys were more likely to report *at least weekly* exposure to sugary beverage advertisements compared to those who were taking all classes at school (AOR = 1.98, 99% CI: 1.27–3.09 and AOR = 1.64, 99% CI: 1.04–2.57, respectively). Respondents in Canada who were taking some classes online/from home were more likely to report *at least weekly* exposure to sugary beverage advertisements compared to those who were taking all classes at school (AOR = 1.30, 99% CI: 1.07–1.56). In Australia, respondents who were taking all classes online/from home were more likely to report *at least weekly* exposure to sugary beverage advertisements compared to those who were taking some classes online/from home (AOR = 1.90, 99% CI: 1.11–3.23) and respondents in Canada were less likely (AOR = 0.77, 99% CI: 0.61–0.97). Sensitivity analyses revealed total screen time was a partial mediator of the association observed and was thus retained in models.

### Setting of exposure to sugary beverage brand advertisements

**Fig. 2:**
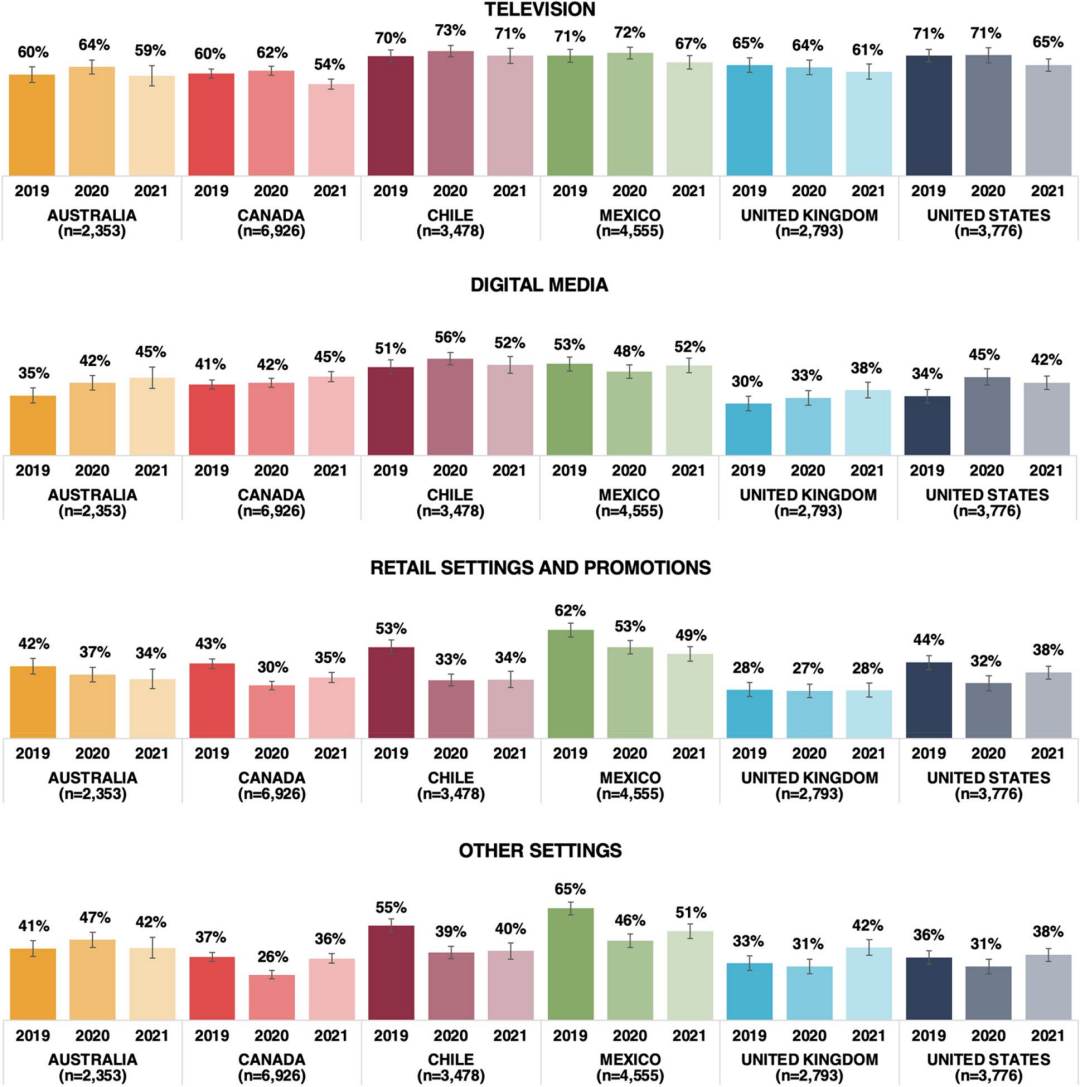
Percentage of respondents reporting exposure to advertisements for any sugary beverage brand^a^ in four settings. Legend: ^a^Reported setting of exposure to Coca-Cola or Red Bull or juice brand were merged to derive the setting of exposure to sugary beverage advertisements in general. *n* = 23 880

The unadjusted weighted percentages of respondents who reported exposure to advertisements for any sugary beverage brand in the past 30 days in four settings by country and year are shown in Fig. 2. Overall, a higher percentage of respondents reported exposure to sugary beverage brand advertisements on television (range: 54%-73%), followed by the other three settings including digital media (range: 30%-56%), retail settings (range: 27%-62%), and other settings (range: 26%-65%), which varied by country and year.

Table 4 shows estimates from separate logistic regression models examining children's self-reported exposure to sugary beverage brand advertisements on television, digital media, retail settings and in other settings in the past 30 days (only among respondents who reported exposure to at least one of the beverage brand advertisements) within countries in 2019, 2020 and 2021. There was a change in reported exposure to sugary beverage brand advertisements on television in Canada only, where respondents were less likely to report exposure in 2021 compared to 2019 and 2020. Respondents in Australia and the US were more likely to report exposure to sugary beverage brand advertisements on digital media in 2020 compared to 2019, and respondents in Australia, Canada, the UK and the US were more likely to report exposure on digital media in 2020 compared to 2019. Respondents in Canada were more likely to report exposure on digital media in 2021 compared to 2020. In Canada, Chile, Mexico and the US, respondents were less likely to report exposure to sugary beverage brand advertisements in retail settings in 2020 and 2021 compared to 2019, and more likely to report exposure in Canada in 2021 compared to 2020. Respondents in Australia were less likely to report exposure in 2021 compared to 2019 in retail settings. Trends in reported exposure to sugary beverage brand advertisements in other settings varied across countries.

**Table 4:** Estimates from logistic regression models examining children's exposure to sugary beverage advertisements in four settings

Parameter	Australia (*n* = 2353)	Canada (*n* = 6926)	Chile (*n* = 3478)	Mexico (*n* = 4555)	United Kingdom (*n* = 2793)	United States (*n* = 3776)
	AOR (99% CI)	AOR (99% CI)	AOR (99% CI)	AOR (99% CI)	AOR (99% CI)	AOR (99% CI)
**Year**						
	**Television**					
2019 vs 2020	1.24 (0.95, 1.62)	1.08 (0.92, 1.26)	1.15 (0.90, 1.47)	1.02 (0.80, 1.31)	0.91 (0.70, 1.19)	1.00 (0.76, 1.33)
2019 vs 2021	0.99 (0.72, 1.36)	0.79 (0.67, 0.93)	1.02 (0.77, 1.35)	0.83 (0.64, 1.07)	0.88 (0.66, 1.16)	0.79 (0.62, 1.00)
2020 vs 2021	0.80 (0.59, 1.09)	0.73 (0.62, 0.86)	0.89 (0.67, 1.16)	0.81 (0.63, 1.04)	0.96 (0.73, 1.26)	0.79 (0.60, 1.03)
	**Digital media**					
2019 vs 2020	1.31 (1.00, 1.71)	1.02 (0.87, 1.19)	1.13 (0.90, 1.42)	0.81 (0.64, 1.01)	1.18 (0.89, 1.56)	1.63 (1.26, 2.11)
2019 vs 2021	1.49 (1.08, 2.06)	1.21 (1.03, 1.43)	1.04 (0.79, 1.35)	0.96 (0.76, 1.22)	1.39 (1.05, 1.84)	1.49 (1.18, 1.89)
2020 vs 2021	1.14 (0.84, 1.54)	1.19 (1.01, 1.41)	0.91 (0.71, 1.18)	1.19 (0.94, 1.50)	1.18 (0.89, 1.54)	0.91 (0.71, 1.18)
	**Retail settings and promotions**					
2019 vs 2020	0.82 (0.63, 1.07)	0.58 (0.49, 0.68)	0.44 (0.35, 0.55)	0.62 (0.49, 0.78)	0.98 (0.74, 1.31)	0.60 (0.46, 0.78)
2019 vs 2021	0.73 (0.53, 1.00)	0.71 (0.60, 0.84)	0.46 (0.35, 0.59)	0.56 (0.44, 0.71)	0.99 (0.74, 1.32)	0.78 (0.62, 0.98)
2020 vs 2021	0.89 (0.65, 1.21)	1.24 (1.04, 1.47)	1.04 (0.80, 1.35)	0.90 (0.72, 1.14)	1.00 (0.75, 1.34)	1.29 (1.00, 1.67)
	**Other settings**					
2019 vs 2020	1.19 (0.92, 1.54)	0.61 (0.52, 0.72)	0.52 (0.42, 0.66)	0.44 (0.35, 0.55)	0.92 (0.69, 1.21)	0.80 (0.62, 1.04)
2019 vs 2021	0.98 (0.71, 1.34)	0.96 (0.81, 1.13)	0.56 (0.43, 0.72)	0.57 (0.45, 0.72)	1.39 (1.06, 1.83)	1.08 (0.86, 1.36)
2020 vs 2021	0.82 (0.61, 1.11)	1.56 (1.31, 1.86)	1.07 (0.83, 1.37)	1.29 (1.02, 1.63)	1.52 (1.16, 2.00)	1.34 (1.04, 1.73)

The year listed first is the reference variable. 'Television' includes television shows, series or movies. 'Digital media' includes websites or social media; video or computer games. 'Retail settings and promotions' includes stores; contests, free samples or coupons; movie theatres. 'Other settings' includes radio; magazines or newspaper; billboard; buses, bus stops and other public transit; school; recreation or community centre; sports event, concert or community event; other. Only among respondents who reported exposure to at least one of the beverage brand advertisements (*n* = 23 880). Models were adjusted for: sex, age category, ethnicity, perceived income adequacy, beverage brand randomization and total screen time (only models with television and digital media as the outcome)

*Abbreviations*: *AOR* Adjusted odds ratio, *CI* confidence interval

### Location of school classes during COVID-19 and setting of exposure to sugary beverage brand advertisements

In separate binary logistic regression models examining the association between location of school classes and setting of exposure to sugary beverage brand advertisements (in 2020 and 2021 only) (Additional file 3), respondents in Australia and the US who were taking all classes online/from home were more likely to report exposure to sugary beverage brand advertisements on television compared to those who were taking some classes online (Australia: AOR = 1.90, 99% CI: 1.10–3.28, US: AOR = 1.68, 99% CI: 1.14–2.48) or all classes at school (Australia: AOR = 1.91, 99% CI: 1.21–3.01, US: AOR = 1.46, 99% CI: 1.02–2.09). Respondents in Canada who were taking all classes online/from home were less likely to report exposure to sugary beverage brand advertisements on television compared to those who were taking some classes online/from home (AOR = 0.75, 99% CI: 0.58–0.97) or all classes at school (AOR = 0.78, 99% CI: 0.62–0.98).

Respondents in Australia and the UK who were taking all classes online/from home at the time of the survey were more likely to report exposure to sugary beverage brand advertisements on digital media compared to those who were taking some classes online/from home (AOR = 2.12, 99% CI: 1.24–3.62, AOR = 2.74, 99% CI: 1.58–4.77, respectively). Respondents in Australia (AOR = 2.98, 99% CI: 1.93–4.60), Canada (AOR = 1.63, 99% CI: 1.29–2.05) and the UK (AOR = 3.33, 99% CI: 2.04–5.44) who were taking all classes online/from home and those who were taking some classes online/from home in Canada (AOR = 1.39, 99% CI: 1.13–1.71) and the US (AOR = 1.55, 99% CI: 1.09–2.19) were more likely to report exposure to advertisements on digital media compared to those who were taking all classes at school. Sensitivity analyses revealed total screen time was a partial mediator of the association observed, thus the model was adjusted for total screen time.

Respondents in Australia (AOR = 2.06, 99% CI: 1.36–3.12), Canada (AOR = 1.52, 99% CI: 1.19–1.95) and the UK (AOR = 2.51, 99% CI: 1.55–4.07) who were taking all classes online/from home at the time of the survey or some classes online/from home (Australia: AOR = 1.78, 99% CI: 1.20–2.63, Canada: AOR = 1.72, 99% CI: 1.37–2.15, UK: AOR = 1.71, 99% CI: 1.21–2.41) were more likely to report exposure to sugary beverage brand advertisements in other settings, compared to those who were taking all classes at school. US respondents who were taking some classes online/from home were more likely to report exposure to sugary beverage brand advertisements in other settings compared to those who were taking all classes at school (AOR = 1.61, 99% CI: 1.14–2.28). There was no association between location of school classes and reporting exposure to sugary beverage brand advertisements in retail settings in any country.

### Exposure to sugary beverage advertisements and sugary beverage intake

**Fig. 3:**
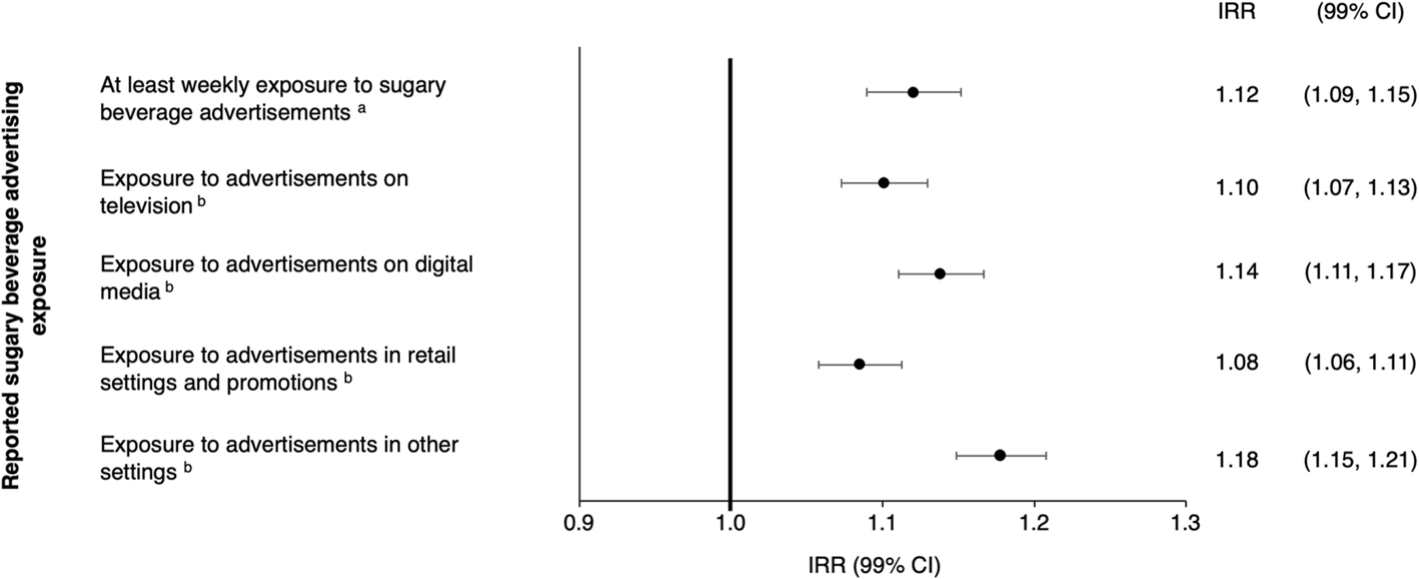
Negative binomial regression model examining the association between children's sugary beverage advertising exposure and intake. Legend: Abbreviations: IRR = incidence rate ratio, CI = confidence interval. Model was adjusted for: country, year, sex, age category, ethnicity, perceived income adequacy, total screen time and beverage brand randomization. ^a^ Versus *less than once a week* exposure; ^b^ Binary setting of advertising exposure variables representing any reported exposure in the past 30 days *vs* no reported exposure. All countries and years. (*n* = 26 465)

Estimates from the negative binomial regression model examining the association between sugary beverage advertisement exposure variables (*at least weekly* exposure; exposure in four settings) and children’s self-reported number of sugary beverages consumed in the past 7 days are shown in Fig. 3. There was an association between all reported advertising exposure variables and reported sugary beverage intake. The number of reported sugary beverages consumed in the past seven days was higher for respondents who reported *at least weekly* exposure to sugary beverage advertisements compared to those who reported *less than once a week* exposure. Exposure to sugary beverage brand advertisements in each of the four settings was associated with a higher sugary beverage intake compared to those who did not report exposure to advertisements in the above settings. Sensitivity analyses revealed that subtracting the number of sugary beverages that may have been sweetened with non-sugar sweeteners from the total number of sugary beverages did not change the pattern of results of the associations between exposure to sugary beverage advertisements and intake among older children, and was thus not accounted for in the statistical model. Location of school classes during the COVID-19 pandemic was not identified as a moderator of the association between reported advertisement exposure variables and sugary beverage intake, although a trend was observed for retail settings (*p* = 0.02).

## Discussion

### Summary of findings

To our knowledge, this is the first study to examine children’s reported exposure to sugary beverage advertisements before and during the COVID-19 pandemic in various countries. Children’s generally stable reported frequency of exposure to sugary beverage advertisements within countries underscores their persistent exposure, even amid the COVID-19 pandemic and the associated changes to children’s living environments. Most children in Australia, Canada, Chile, Mexico, the UK and the US reported seeing sugary beverage advertisements at least once a week, with little variation over time within countries. In most countries, there was an increase in reported exposure to sugary beverage advertisements on digital media from 2019 to 2021, with a concomitant decrease in retail settings. Attending all or some school classes online during the COVID-19 pandemic was associated with greater likelihood of exposure to sugary beverage advertisements in some countries, compared to attending in-person school classes. Children who reported frequent exposure to sugary beverage advertisements and exposure to sugary beverage advertisements in each setting were more likely to report higher intake of sugary beverages.

### Comparison to previous research and interpretation of findings

High percentages of children in all countries reported *at least weekly* exposure to sugary beverage advertisements, with a *few times a week* being the most frequent selected response, which is consistent with prior research using objective marketing exposure data. A study examining children’s exposure to less healthy food marketing using wearable devices in New Zealand estimated exposure 27 times per day on average across various settings (*e.g*. food packaging, signs, school), which is likely an underestimate on screen-based media (*e.g*., smart phones, tablets, computers) due to methodological constraints [2]. Studies in Canada, Australia and Mexico examining digital media advertising exposure estimated that children were exposed to advertising for less healthy foods 30 to 189 times per week [3, 42, 43]. Importantly, findings from our study underestimate children’s exposure to overall less healthy food advertising, as exposure to sugary beverage advertisements was assessed specifically.

Respondents’ greater reporting of sugary beverage advertisements on television, followed by the other three settings including digital media, retail settings, and other settings is consistent with previous studies [37, 44], and may be attributed to the distinct types of advertising content employed across these settings. Television advertising may be more easily identifiable compared to advertising content on digital media [45, 46], where subtle and varied techniques are often embedded in other web content, such as celebrity-generated content [3]. Despite a high percentage of respondents reporting advertising exposure on digital media, this is likely an underestimation of actual exposure. Advertising can influence children’s behaviours even without conscious perception of exposure [47], which is particularly important for digital media, where children are extensively exposed to powerful persuasive techniques such as digital game-based and influencer advertising [48]. Advertising content on digital media may also be more targeted by using cookies that record personal preferences to personalise content to individual users [12], which may further enhance the power of the advertising messages [11].

Despite changes in lifestyles during the COVID-19 pandemic, children were exposed to similar levels of advertising, likely due to changes in food companies' advertising strategies. Reported frequency of exposure to sugary beverage advertisements was relatively stable across years, but there were changes in the settings of exposure, with notable increases on digital media, and decreases in retail settings across years. Media consumption habits are shifting globally from traditional (*e.g.,* television) to digital media channels [49, 50], and companies are adapting their marketing strategies accordingly. Digital media advertising expenditures have increased in recent years [51], although still not as high as expenditures on traditional media [19, 20], in part due to underestimation of costs and the lower cost of digital media advertising [52]. The increase in respondents’ reported exposure to sugary beverage advertising on digital media in 2021 compared to 2019 in most countries was independent of total screen time, suggesting that factors other than changes in media consumption habits during the COVID-19 pandemic influenced exposure to digital advertising. This may reflect an acceleration of the digitalisation of marketing during the COVID-19 pandemic [53], although an increasing trend was likely already underway. It is not clear whether this is a temporary increase associated with the COVID-19 pandemic or if increased exposure will remain stable or increase further. The concomitant decrease in reported advertising exposure in retail settings may be due to reduced access to retail settings amid COVID-19 containment and closure measures [35], but may also reflect a decrease in advertising efforts in this setting.

These results may reflect the influence of containment and closure measures during the COVID-19 pandemic which may have unintendedly increased children’s exposure to sugary beverage advertisements. Children who were taking all classes online may have had more time for other activities, such as playing video games [54] or browsing social media, which may partly explain their greater likelihood of reporting frequent exposure to advertisements and of reporting exposure to sugary beverage advertisements on digital media and in other settings (*e.g.,* magazines, radio) in some countries, compared to children attending all classes in-person.

This study suggests that children’s frequent reported exposure to sugary beverage advertisements in multiple settings may be influencing children’s sugary beverage consumption patterns, consistent with previous research [5, 6, 9, 55]. Even a low threshold of *at least weekly* exposure to sugary beverage advertising was associated with sugary beverage intake, as was the reported exposure to all the individual media and settings. While the repeated cross-sectional design of our study cannot confirm a causal link between advertising exposure and intake of sugary beverages, previous studies provide evidence supporting a causal relationship between children’s exposure to food advertising and consumption behaviours [56].

### Policy implications and future research

The findings of this research suggest that reducing children’s exposure to sugary beverage advertising across all settings, including on digital media, may help reduce children’s sugary beverage intake. This aligns with recent WHO recommendations for comprehensive regulations to restrict marketing of less healthy foods in all media and settings in which children may be exposed to support healthy child development [11].

The results suggest that the existing regulatory frameworks in these countries, which include a mix of industry-led self-regulatory approaches and governmental regulatory strategies, were not sufficient to reduce children’s exposure to the advertising of less healthy beverages. This may be explained by a focus on restricting the advertising of less healthy food products and neglecting restrictions of brand advertising, a common advertising strategy [57], which may be particularly powerful for widely recognized beverage brands. This may also reflect loopholes in current regulations [58], such as a displacement of less healthy food advertising from regulated media or settings (*e.g.,* television) to unregulated settings (*e.g.,* digital media) [33].

Recent policy guidelines emphasize extending marketing restrictions beyond child-directed settings [11]. Children are frequently exposed to advertising in settings that are not specifically targeted to them (*e.g.,* social media) [3], and often not included in child-directed marketing restrictions [33]. Previous evidence has suggested that broader restrictions, such as Chile’s time-based advertising ban from 6 a.m. to 10 p.m. (implemented in 2018) were more effective in reducing children’s exposure to less healthy food advertising on television than child-directed approaches alone [59]. While our study did not find that children in Chile reported less exposure to sugary beverage advertisements compared to other countries, notable reductions in *at least weekly* exposure across study waves were observed. Our study does not have pre-implementation data to examine whether exposure was higher before such a policy was in place.

The findings highlight children’s increased reported exposure to sugary beverage advertising during COVID-19 pandemic school closures in some countries, which may help inform future health crises by ensuring public health measures implemented are supported by efforts to protect vulnerable populations from unintended policy consequences. Future research should examine children’s exposure to less healthy food advertising more broadly (*i.e.,* not only for sugary beverages) during years affected by the COVID-19 pandemic, and monitor exposure in various settings, with a focus on digital media given the increasing trend in reported exposure over time.

### Strengths and limitations

This study has a large sample size, and the use of the same measures across countries and years allows for comparisons within and between countries. Unlike many studies that focus on a single setting of advertising exposure (*i.e.,* television or digital media) [10], this study examined exposure across various settings. The use of self-reported advertising exposure data allowed for examination of individual-level exposure and correlates, such as location of school classes during the COVID-19 pandemic. Although likely an underestimation of actual exposure to advertising given the implicit nature of advertising [1], self-reported advertising exposure measures have been used in large population samples [8, 37, 44, 60] and show high correlation with objective exposure data [61, 62].

Respondents were recruited using non-probability based sampling; thus, the findings do not provide nationally representative estimates. However, the data were weighted, which should help mitigate this. Self-reported measures may create recall and social desirability bias, although the latter may be reduced by the use of online surveys [63]. The study’s focus on the promotional (advertising) aspect of marketing is a limitation, as studying marketing as an integrated set of activities (*i.e.,* including product, price, placement), may help to develop more effective marketing regulations [64]. Respondents’ interpretation of what constitutes an advertisement may further introduce bias. Finally, children aged 14–17 years were asked to report the proportion of four beverage categories that were ‘diet, low-calorie or no-calorie’; 38% of sports drinks, 33% of energy drinks, 23% of fruit drinks and 53% of flavoured waters were ‘diet, low-calorie or no-calorie’, and this was not accounted for in analyses. Thus, there may be an overestimation of sugary beverage intake for some participants, but differences in estimation would be expected to be similar over time, and it is expected that children interpreted the question similarly across countries.

## Conclusion

This study demonstrated increased reported exposure of children to sugary beverage digital advertising from 2019 to 2021 in most countries, which was associated with greater intake of sugary beverages, independent of screen time. Effective marketing restrictions that reduce children’s exposure to sugary beverage advertisements, including on digital media, may be one means to improve population-level diets and reduce consumption of sugary beverages.

## Supplementary Information

Supplementary Material 1.

Supplementary Material 2.

Supplementary Material 3.

## Data Availability

The datasets used and analysed during the current study are available from the corresponding author on reasonable request.

## Abbreviations

UK: United Kingdom
US: United States
WHO: World Health Organization
IFPS: International Food Policy Study
BFQ: Beverage Frequency Questionnaire
AOR: Adjusted odds ratio
IRR: Incidence rate ratio
CI: Confidence interval
