# Supplementary material for: Children’s self-reported exposure to sugary beverage advertisements and association with intake across six countries before and during the COVID-19 pandemic: a repeat cross-sectional study
Source: BMC Public Health. 2024 Oct 11;24:2787. doi: 10.1186/s12889-024-20210-8 (PMC11470686; doi:10.1186/s12889-024-20210-8)
Supplement: Supplementary file 1 — Supplementary Material 1. [file 12889_2024_20210_MOESM1_ESM.pptx]

## Slide 1
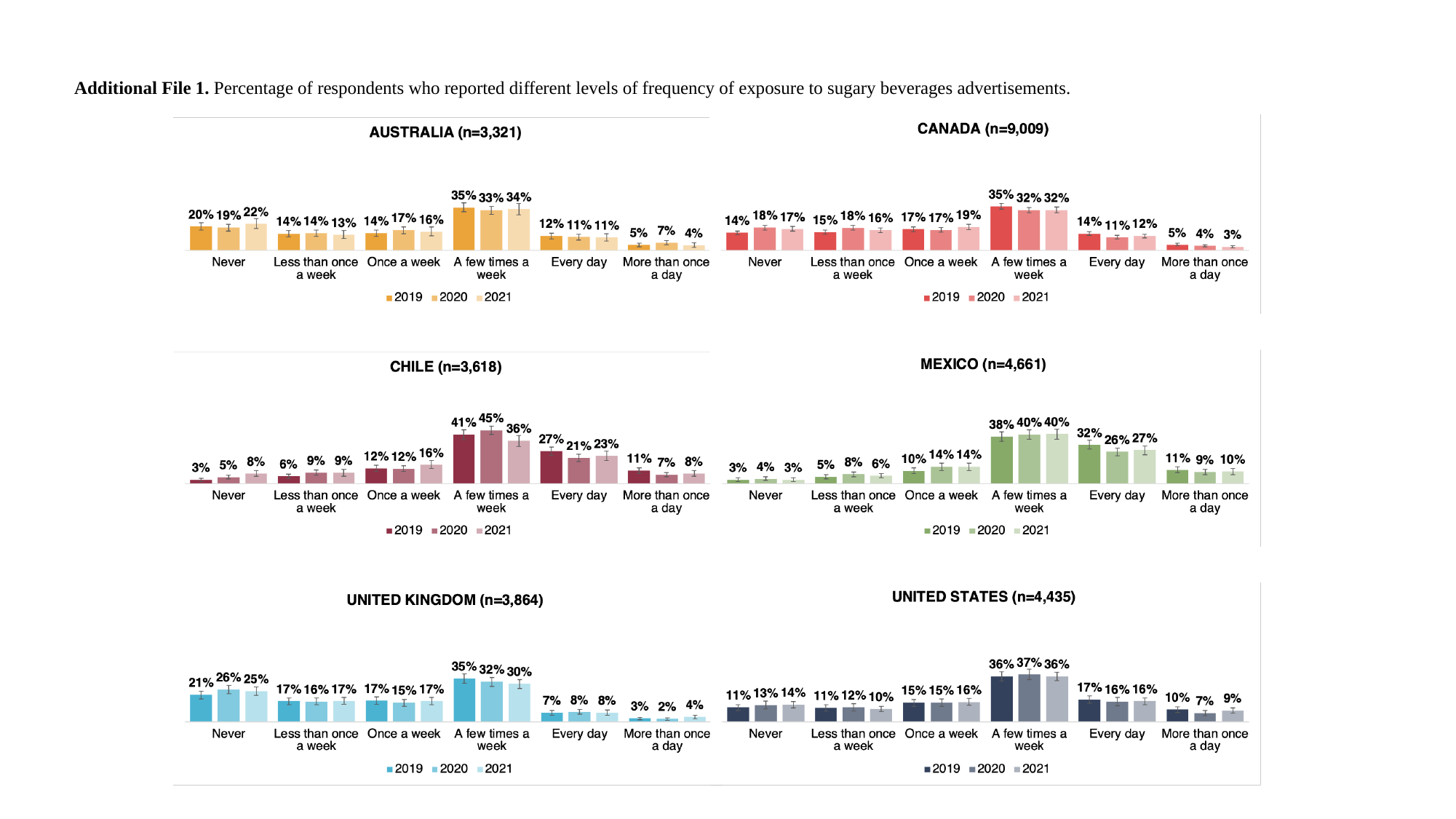

Additional File 1. Percentage of respondents who reported different levels of frequency of exposure to sugary beverages advertisements.
