# Supplementary material for: Children’s self-reported exposure to sugary beverage advertisements and association with intake across six countries before and during the COVID-19 pandemic: a repeat cross-sectional study
Source: BMC Public Health. 2024 Oct 11;24:2787. doi: 10.1186/s12889-024-20210-8 (PMC11470686; doi:10.1186/s12889-024-20210-8)
Supplement: Supplementary file 2 — Supplementary Material 2. [file 12889_2024_20210_MOESM2_ESM.pptx]

## Slide 1
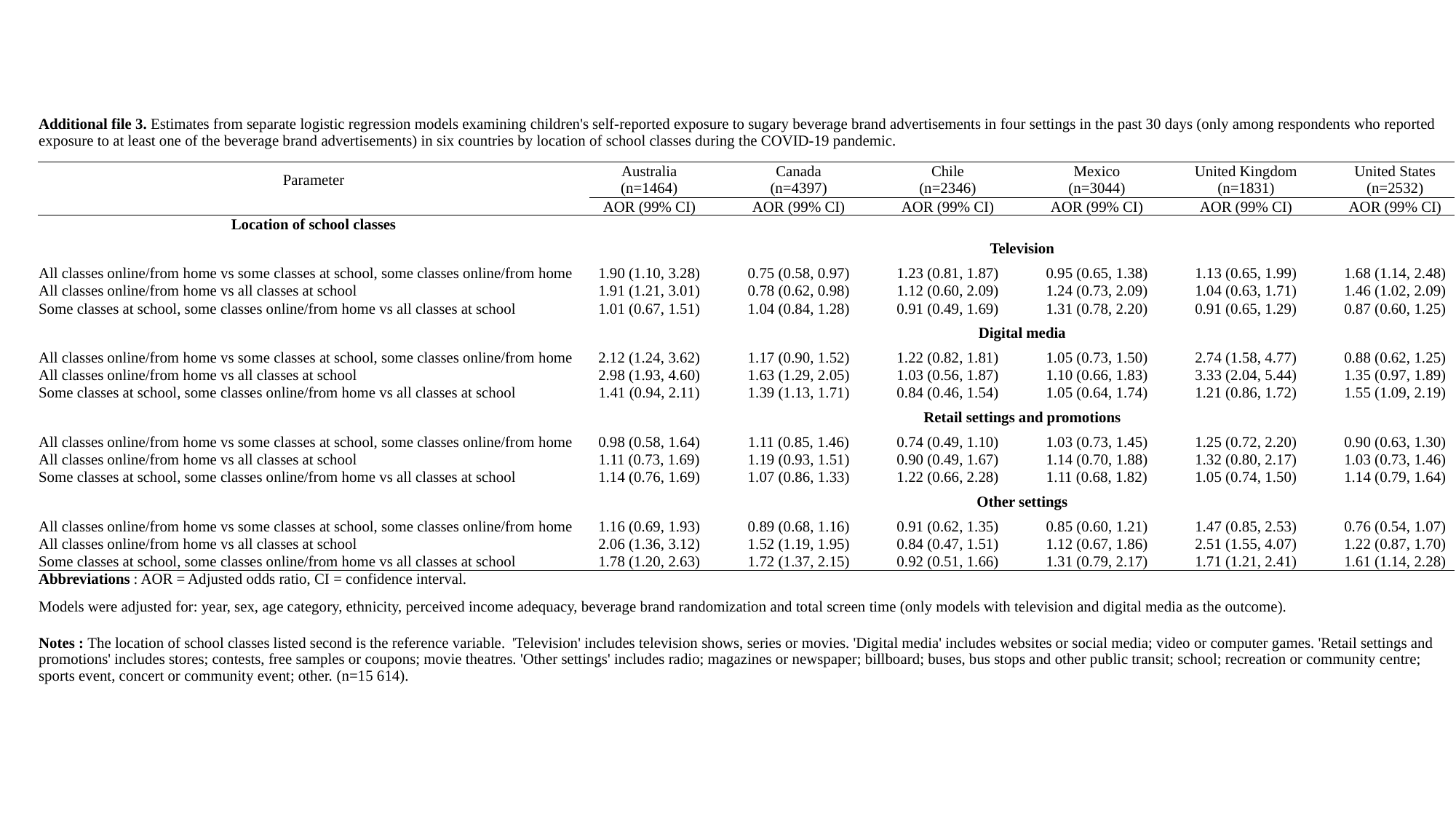

| Additional file 3. Estimates from separate logistic regression models examining children's self-reported exposure to sugary beverage brand advertisements in four settings in the past 30 days (only among respondents who reported exposure to at least one of the beverage brand advertisements) in six countries by location of school classes during the COVID-19 pandemic. | | | | | | | | | | | |
| --- | --- | --- | --- | --- | --- | --- | --- | --- | --- | --- | --- |
| Parameter | Australia (n=1464) | | Canada (n=4397) | | Chile (n=2346) | | Mexico (n=3044) | | United Kingdom (n=1831) | | United States (n=2532) |
| | AOR (99% CI) | | AOR (99% CI) | | AOR (99% CI) | | AOR (99% CI) | | AOR (99% CI) | | AOR (99% CI) |
| Location of school classes | | | | | | | | | | | |
| | Television | | | | | | | | | | |
| All classes online/from home vs some classes at school, some classes online/from home | 1.90 (1.10, 3.28) | | 0.75 (0.58, 0.97) | | 1.23 (0.81, 1.87) | | 0.95 (0.65, 1.38) | | 1.13 (0.65, 1.99) | | 1.68 (1.14, 2.48) |
| All classes online/from home vs all classes at school | 1.91 (1.21, 3.01) | | 0.78 (0.62, 0.98) | | 1.12 (0.60, 2.09) | | 1.24 (0.73, 2.09) | | 1.04 (0.63, 1.71) | | 1.46 (1.02, 2.09) |
| Some classes at school, some classes online/from home vs all classes at school | 1.01 (0.67, 1.51) | | 1.04 (0.84, 1.28) | | 0.91 (0.49, 1.69) | | 1.31 (0.78, 2.20) | | 0.91 (0.65, 1.29) | | 0.87 (0.60, 1.25) |
| | Digital media | | | | | | | | | | |
| All classes online/from home vs some classes at school, some classes online/from home | 2.12 (1.24, 3.62) | | 1.17 (0.90, 1.52) | | 1.22 (0.82, 1.81) | | 1.05 (0.73, 1.50) | | 2.74 (1.58, 4.77) | | 0.88 (0.62, 1.25) |
| All classes online/from home vs all classes at school | 2.98 (1.93, 4.60) | | 1.63 (1.29, 2.05) | | 1.03 (0.56, 1.87) | | 1.10 (0.66, 1.83) | | 3.33 (2.04, 5.44) | | 1.35 (0.97, 1.89) |
| Some classes at school, some classes online/from home vs all classes at school | 1.41 (0.94, 2.11) | | 1.39 (1.13, 1.71) | | 0.84 (0.46, 1.54) | | 1.05 (0.64, 1.74) | | 1.21 (0.86, 1.72) | | 1.55 (1.09, 2.19) |
| | Retail settings and promotions | | | | | | | | | | |
| All classes online/from home vs some classes at school, some classes online/from home | 0.98 (0.58, 1.64) | | 1.11 (0.85, 1.46) | | 0.74 (0.49, 1.10) | | 1.03 (0.73, 1.45) | | 1.25 (0.72, 2.20) | | 0.90 (0.63, 1.30) |
| All classes online/from home vs all classes at school | 1.11 (0.73, 1.69) | | 1.19 (0.93, 1.51) | | 0.90 (0.49, 1.67) | | 1.14 (0.70, 1.88) | | 1.32 (0.80, 2.17) | | 1.03 (0.73, 1.46) |
| Some classes at school, some classes online/from home vs all classes at school | 1.14 (0.76, 1.69) | | 1.07 (0.86, 1.33) | | 1.22 (0.66, 2.28) | | 1.11 (0.68, 1.82) | | 1.05 (0.74, 1.50) | | 1.14 (0.79, 1.64) |
| | Other settings | | | | | | | | | | |
| All classes online/from home vs some classes at school, some classes online/from home | 1.16 (0.69, 1.93) | | 0.89 (0.68, 1.16) | | 0.91 (0.62, 1.35) | | 0.85 (0.60, 1.21) | | 1.47 (0.85, 2.53) | | 0.76 (0.54, 1.07) |
| All classes online/from home vs all classes at school | 2.06 (1.36, 3.12) | | 1.52 (1.19, 1.95) | | 0.84 (0.47, 1.51) | | 1.12 (0.67, 1.86) | | 2.51 (1.55, 4.07) | | 1.22 (0.87, 1.70) |
| Some classes at school, some classes online/from home vs all classes at school | 1.78 (1.20, 2.63) | | 1.72 (1.37, 2.15) | | 0.92 (0.51, 1.66) | | 1.31 (0.79, 2.17) | | 1.71 (1.21, 2.41) | | 1.61 (1.14, 2.28) |
| Abbreviations : AOR = Adjusted odds ratio, CI = confidence interval. | | | | | | | | | | | |
| Models were adjusted for: year, sex, age category, ethnicity, perceived income adequacy, beverage brand randomization and total screen time (only models with television and digital media as the outcome). | | | | | | | | | | | |
| Notes : The location of school classes listed second is the reference variable.  'Television' includes television shows, series or movies. 'Digital media' includes websites or social media; video or computer games. 'Retail settings and promotions' includes stores; contests, free samples or coupons; movie theatres. 'Other settings' includes radio; magazines or newspaper; billboard; buses, bus stops and other public transit; school; recreation or community centre; sports event, concert or community event; other. (n=15 614). | | | | | | | | | | | |
