# Supplementary material for: Children’s self-reported exposure to sugary beverage advertisements and association with intake across six countries before and during the COVID-19 pandemic: a repeat cross-sectional study
Source: BMC Public Health. 2024 Oct 11;24:2787. doi: 10.1186/s12889-024-20210-8 (PMC11470686; doi:10.1186/s12889-024-20210-8)
Supplement: Supplementary file 3 — Supplementary Material 3. [file 12889_2024_20210_MOESM3_ESM.pptx]

## Slide 1
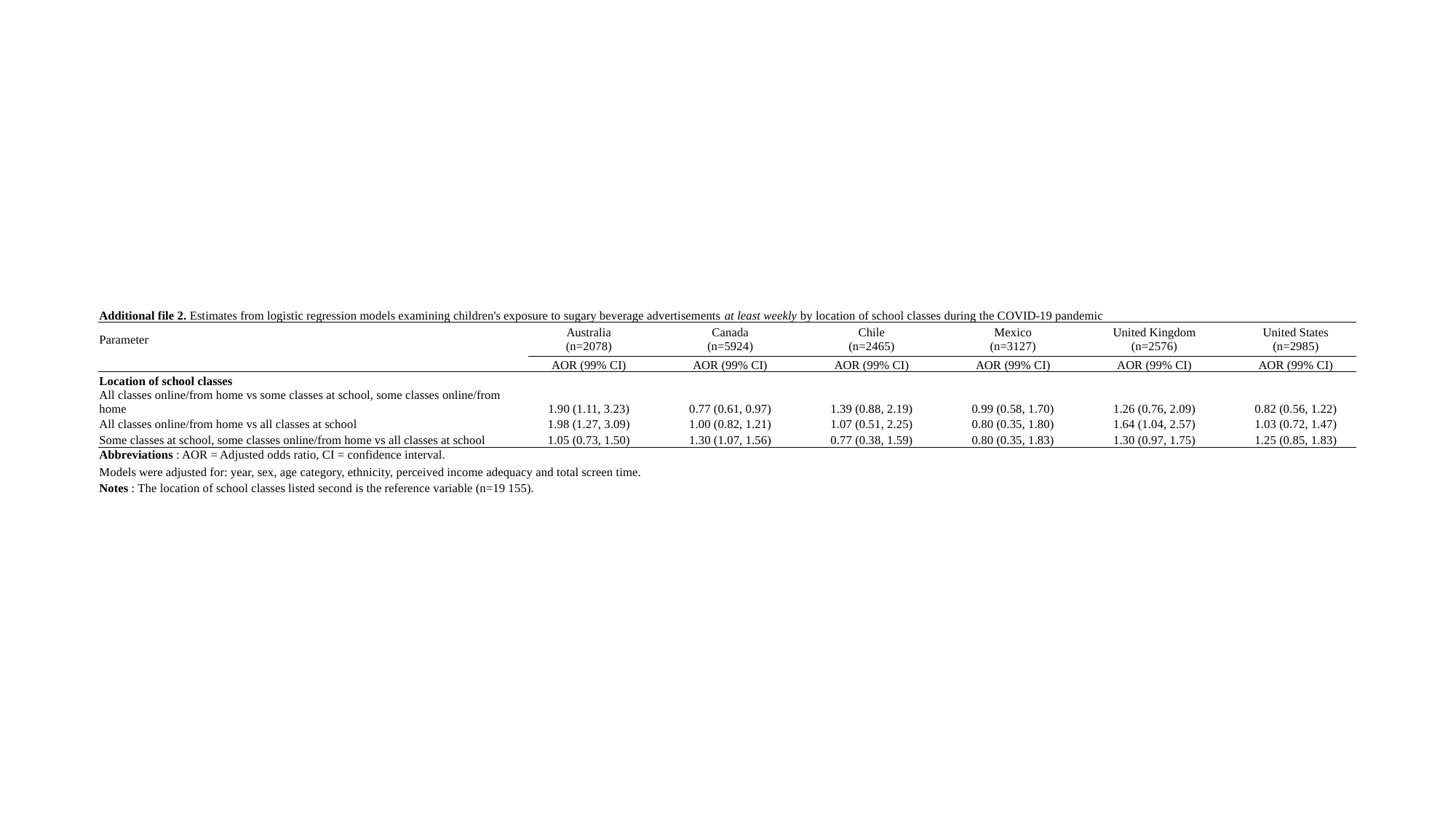

| | | | | | | | | | | | |
| --- | --- | --- | --- | --- | --- | --- | --- | --- | --- | --- | --- |
| | | | | | | | | | | | |
| Additional file 2. Estimates from logistic regression models examining children's exposure to sugary beverage advertisements at least weekly by location of school classes during the COVID-19 pandemic | | | | | | | | | | | |
| Parameter | Australia (n=2078) | | Canada (n=5924) | | Chile (n=2465) | | Mexico (n=3127) | | United Kingdom (n=2576) | | United States (n=2985) |
| | AOR (99% CI) | | AOR (99% CI) | | AOR (99% CI) | | AOR (99% CI) | | AOR (99% CI) | | AOR (99% CI) |
| Location of school classes | | | | | | | | | | | |
| All classes online/from home vs some classes at school, some classes online/from home | 1.90 (1.11, 3.23) | | 0.77 (0.61, 0.97) | | 1.39 (0.88, 2.19) | | 0.99 (0.58, 1.70) | | 1.26 (0.76, 2.09) | | 0.82 (0.56, 1.22) |
| All classes online/from home vs all classes at school | 1.98 (1.27, 3.09) | | 1.00 (0.82, 1.21) | | 1.07 (0.51, 2.25) | | 0.80 (0.35, 1.80) | | 1.64 (1.04, 2.57) | | 1.03 (0.72, 1.47) |
| Some classes at school, some classes online/from home vs all classes at school | 1.05 (0.73, 1.50) | | 1.30 (1.07, 1.56) | | 0.77 (0.38, 1.59) | | 0.80 (0.35, 1.83) | | 1.30 (0.97, 1.75) | | 1.25 (0.85, 1.83) |
| Abbreviations : AOR = Adjusted odds ratio, CI = confidence interval. | | | | | | | | | | | |
| Models were adjusted for: year, sex, age category, ethnicity, perceived income adequacy and total screen time. | | | | | | | | | | | |
| Notes : The location of school classes listed second is the reference variable (n=19 155). | | | | | | | | | | | |
